# Bored, Distracted, and Confused: Emotions That Promote Creativity and Learning in a 28-Month-Old Child Using an iPad

**DOI:** 10.3390/jintelligence10040118

**Published:** 2022-12-02

**Authors:** Shiva Khalaf, Hechmi Kilani, Melissa B. Razo, Elena L. Grigorenko

**Affiliations:** Department of Psychology, University of Houston, Houston, TX 77004, USA

**Keywords:** digital game-based learning, mind-wandering, preschool children, creativity, creative problem solving, learning

## Abstract

Digital technology is increasingly becoming a part of daily life, including the lives of children. Portable digital devices are omnipresent and integrated into activities that did not previously require them. The related skills are often referred to as 21st-century skills, constituting a new type of literacy: digital literacy. These devices and skills bring unique, innovative elements to the learning experience; yet, we do not know the extent to which behavior, emotion, and socialization are affected by such experience. For preschool-aged children, interactions with digital devices and games for the purposes of learning can lead to a state of confusion and boredom, an emotional driving force that may generate mind-wandering and exploration, which, in turn, may facilitate learning. Our interdisciplinary observational case study examined the behavioral patterns linked to digital game-based learning (DGBL) by observing how a child’s mind-wandering contributed to iPad use when they were allowed to freely engage with the device and explore independently during the learning process. Building on a previous case study of a 28-month-old boy, “Ryan”, we evaluated the effects of bouts of mind-wandering as he played various DGBL applications (apps) by examining the length of time that Ryan exhibited relevant affective and behavioral states, iPad manipulations, and social interaction during the playtime. Ryan’s interactions with the iPad were video recorded for five weeks, and the video footage was coded using a detailed rubric. The results indicated that negative emotions, such as boredom, distraction, and confusion, if coupled with attentiveness and persistence, led to positive mind-wandering and positive learning outcomes. However, when boredom was coupled with frustration, it led to negative mind-wandering and a lack of learning outcomes. In conclusion, our study presents evidence that DGBL apps may improve learning by capitalizing on positive and avoiding negative mind-wandering.

## 1. Introduction

The American Academy of Pediatrics (AAP) has several recommendations regarding screen time for children aged five and below. These recommendations encompass an array of media devices: televisions, computers, and mobile devices. It is advised that parents not allow a child between the ages of two and five more than one hour per day in front of a screen. This one hour should consist of high-quality programming—which generally means the programming should be educational in nature—and should be consumed while in the company of a caregiver (Council on Communications and Media 2016). The AAP has also acknowledged that new evidence is coming forth which suggests that interactive applications (apps) used on touchscreen devices may be effective educational tools for children aged 24 months or older. However, this research was carried out with unique, experimental apps not available to the public (Council on Communications and Media 2016). The COVID-19 pandemic introduced a situation within education that was not foreseen, and its duration has brought to the attention of educators and parents how needed digital literacy, a 21st-century skill, and effective digital education opportunities are for children (UNESCO 2021). In light of this recent experience and the AAP’s finding regarding interactive apps, we wanted to investigate whether digital game-based learning (DGBL) via the currently available interactive iPad apps exhibits the same efficacy as the experimental apps. In studying this, we hoped to uncover that DGBL offers a learning benefit to children in today’s changing, 21st-century technological environment and that this benefit should be capitalized on in preparation for the new natural and societal challenges.

The use of digital media has been reported to have both negative and positive outcomes. With regard to the former, excessive media use has been associated with poor motor skills and increased physical inactivity (Felix et al. 2020), decreased attention (Vigil 2019), unfavorable psychological outcomes with regard to mental health, cognitive functioning, and academic achievement, and poor language development (Duch et al. 2013; Oswald et al. 2020). Such findings possibly stem from a lack of child–parent interaction and reduced quantity and quality of playtime for these children, especially since a child’s indirect interactions with an adult caregiver, such as praise, indirect commands, and questions from the caregiver, have been shown to boost their self-esteem, exploration, and creativity (Bonnette et al. 2001).

Regarding the latter, one possible benefit from DGBL could be the freedom to engage in mind-wandering—those moments when attention shifts away from the task at hand and onto something unrelated. Mind-wandering may provide a child time and space to engage in creative problem solving, also referred to as a time of creative incubation (Baird et al. 2012), that enhances their ability to learn. Behnamnia et al. (2020a) conducted a review of 20 published articles to determine if the studies presented evidence of positive outcomes in children ages three to six who engaged with DGBL. They found several positive learning outcomes such as creativity (defined in various ways, including finding creative solutions to problems and creative play), self-efficacy with the technology, increased critical thinking skills, and better learning performance (Behnamnia et al. 2020a). The authors observed that the use of digital games, particularly on a mobile device, could increase learning in multiple disciplines and academic subject areas from science/math to the liberal arts by using techniques that allow children to visualize the concepts in everyday life, such as the ability to “see” invisible abstracts, or engaging children through attractive means like storytelling (ibid.). Another study demonstrated that mind-wandering provided an incubation period for the participants by having them attempt to solve the same problem set before and after allowing the mind to wander (Baird et al. 2012). Among the four experimental conditions, it was shown that the group who engaged in an undemanding task—in this case, “… a choice reaction time task (0-back) requiring infrequent responses” (ibid.)—scored higher in the second round of testing than the other groups, providing evidence that when a person’s attention is moved away from the task at hand and toward something that allows them to mind-wander, the person is prone to creative incubation (ibid.).

In an attempt to elucidate contradictory findings, the helpfulness and adaptability of mind-wandering and its derived costs and benefits were reviewed by Mooneyham and Schooler (2013). While they found that mind-wandering has been observed to have negative effects on mood, reading comprehension, sustained attention, and certain other cognitive functions, they observed that it has also had positive outcomes (ibid.). Based on the compiled literature, it appeared that while mind-wandering can detract from certain performance factors such as the ones listed above, it benefits creative thinking and dishabituation from the task. It has been stated that these processes, in turn, can lead to enhanced learning.

According to this synthesis of the literature, it is possible that there are many benefits to be derived for young children’s learning while using DGBL apps on a mobile device when these apps allow a child to engage in mind-wandering that leads to creative problem solving. However, no child under the age of three was tested or observed by any of them. Additionally, only one of the studies pointed to self-efficacy with digital technology as a measure of learning while using numerous academic or cognitive measures (Behnamnia et al. 2020a). The ultimate goal of learning is to obtain and increase skills (academic as well as others), but if a child is to learn those skills on a digital platform, it is important that they are able to also learn how to navigate the platform itself. The creative incubation triggered or facilitated by moments of mind-wandering may help a child to learn, not only the academic material being presented, but how to effectively navigate the digital environment in which the material is being presented. To our knowledge, there have not been any empirical studies attempting to combine the two conditions, i.e., allowing the mind to wander while using a DGBL app. As stated above, the COVID-19 pandemic exemplifies one of the challenges of the 21st century, and we believe that it will greatly benefit children to become proficient in using DGBL apps. Correspondingly, the field of cognitive psychology and related fields need to understand the balance of benefits and obstacles related to this form of learning. Therefore, there is a concern about the lack of studies on mind-wandering and its relationship to preschool-aged children’s use of DGBL apps. 

Our current study is the second in a series of case studies with a participant named “Ryan.” At the time of the data collection, Ryan was a 28-month-old child for whom this was the first systematic iPad exposure. In the first study (Zhukova et al. 2020), Ryan was given several opportunities over the course of five weeks to play DGBL apps of his choice in the presence of one or more caregivers. These play sessions were video recorded, and the recordings were coded for affects, behaviors, verbalizations, and iPad manipulations displayed by Ryan. These data were combined with his performance outcomes on the app he chose to play most often, *Doodle Dots*, to identify relationships between his affects and learning performance, defined as his acquired digital literacy and academic performance. The recordings were also coded for caregiver verbalizations to explore the roles of a caregiver and of social interactions in digital literacy acquisition and learning. The findings indicated that Ryan increased his speed and manipulation proficiency in using the iPad over the period of five weeks. The frustration he displayed during use correlated to a negative mind-wandering outcome—an increase in the number of errors he made—while displays of attentiveness, help-seeking, and persistence correlated to a positive mind-wandering outcome: his generation of novel situations and increased creativity.

Our study further investigated how the use of a DGBL app can contribute to a child’s learning, defined as the development of app navigation skills and effective iPad manipulations, and how mind-wandering is a positive contribution to that learning. While mind-wandering is an abstract mental activity, in psychological research, it has been operationalized as task-unrelated thought (Murray and Krasich 2022). In the current study, this construct was quantified as moments when Ryan shifted his attention away from the task at hand and onto something unrelated as a result of his boredom, distraction, and/or confusion. Past studies have shown that if such distractions and task-unrelated thoughts are coupled with attentiveness and persistence, they could lead to positive mind-wandering that facilitates creative problem solving and greater learning (Belton and Priyadharshini 2007; Bonnette et al. 2001; Kort et al. 2001; Pachai et al. 2016; Zhukova et al. 2020). Conversely, if the tendency to shift attention from the here-and-now is combined with frustration, it could lead to negative mind-wandering and a lack of positive learning outcomes (Kort et al. 2001; Maloney et al. 2015; Pachai et al. 2016; Zhukova et al. 2020). Because past studies have used self-reporting by the participants to measure the amount of mind-wandering, and because a child at this age cannot effectively carry that out, we gauged his bouts of mind-wandering as he used the game apps by recording the moments of boredom or distraction as per an affect coding rubric we developed. These affects were coded for frequency during the first study, but during the second study, we coded for the duration of time the child exhibited such behavior. Additionally, in this rendition of the data, Ryan was observed as he played several different apps on the iPad, not only *Doodle Dots*. Our hypothesis was that, as he played these interactive, educational game apps on the iPad, he would engage in mind-wandering, and this mind-wandering would lead to periods of creative incubation that would mediate positive learning outcomes.

## 2. Materials and Methods

### 2.1. Participant 

Ryan is a 28-month-old Caucasian male raised in a bilingual, Russian–English household. He began attending preschool at 18 months of age. Our study gathered observational data on Ryan while he was in the care of a childcare provider (hereafter, caregiver) three times per week for five weeks, which resulted in 15 timepoints of data collection. Ryan is a typically developing child; for more information regarding Ryan’s developmental skills, please see Zhukova et al. (2020). The study was approved by the Yale IRB. 

### 2.2. iPad Apps Used

*Doodle Dots* (PBS KIDS Sprout 2011) is a game targeted at preschool children. In this application, children respond to verbal prompts to connect dots and complete a picture. The “dots” the child is required to connect are either numbers, colors, geometric shapes, or fruit. A child can select their choice of dots from icons at the top of the screen and can select the picture puzzle they wish to complete from a gallery of images, which includes animals, toys, foods, vehicles, etc. If the child selects the wrong dot after a prompt, they will receive corrective feedback (e.g., “That’s not a green dot!”). There is no time limit or limit to the number of incorrect responses a child can receive.

*ABC Go* (Peapod Labs 2010) focuses on exposing children to a variety of vehicles and methods of transportation by exploring the alphabet and linking letters to words. This application teaches children new words through sight, sound, and touch. After choosing a letter icon, a child is exposed to a means of transportation that begins with that specific letter. The child can also hear a description of the vehicle, see a video of the vehicle in use, or play a puzzle in which they must “find” the vehicle by filling in its outline as delineated by a dashed line. 

*Memory King* (Innovative Mobile Apps 2011) is a card-matching game that seeks to reinforce memory skills and develop vocabulary. The child chooses the number of cards to be matched, which can range from 2 to 32 pairs. The categories of cards utilized in this study include animals, numbers, letters, shapes, and colors. 

*Monkey Preschool Lunchbox* (THUP Games 2009) features seven minigames within the context of a monkey trying to fill his lunch box. After every few rounds of the game, a child is rewarded with a virtual sticker that they can drag onto a bulletin board. None of the games have time limits, and the monkey in the corner provides positive and negative feedback with respect to how the child responds to a given prompt. 

*Memory Train* (Piikea St. LLC 2011) presents its memory development activities in an adaptive play environment. It uses a variety of objects that test the memory of colors, facial expressions, and specific aspects of larger pictures.

*Agnitus’* (Agnitus 2012) multitude of activities covers 22 different academic skills based on the Common Core State Standards. This game allows a child to master a variety of skills, such as the recognition of colors and shapes, basic counting and sorting, and matching objects and letters.

*Counting Ants Lite* (Playtend Apps LLP 2009) helps young children learn the numbers one through ten. The app cycles through a series of scenes and minigames in which a gradually increasing number of cars encounters obstacles along its drive. The app seeks to introduce children to the concept of quantity in a variety of ways. There are no auditory instructions, and while there is text onscreen indicating what to do at each point in the game, a child need not know how to read to follow the intuitive style of play.

### 2.3. Study Design 

Ryan was presented with an iPad. Without receiving any instruction on how to operate the device, he was allowed to play the DGBL apps available on it. Each play session lasted an average of 25 min, and Ryan’s behavioral interactions with the iPad and his caretaker were video recorded. This occurred over the course of 5 weeks for a total of 6:27:33 hours of footage. The video recordings were then observed and coded for Ryan’s affects, behaviors, and iPad manipulations, as well as for the verbal interactions that occurred between him and the caregiver.

#### 2.3.1. Experimental Setting

The experimental setting was a naturalistic setting, in the home of Ryan’s day caregiver. Both he and the experimental caregiver sat at a quiet table and interacted with the iPad. The setting was not controlled or altered in any way besides minimizing interactions with members of the household during play. Experimental priority was placed on the dyad, Ryan and his caregiver. 

#### 2.3.2. Coding Scheme

Behavioral coding of the video footage was conducted by seven undergraduate volunteers working under the supervision of a graduate student for a total of eight coders. The undergraduate coders were trained by the supervisor for approximately 2 months on the coding process and proper use of the coding rubric. During this time, the coders met one or two times per week to compare their practice codes, calibrate them, and refine the rubric.

After training, the 15 recordings were divided into two groups, and four coders were randomly assigned to each group. Each recording was randomly assigned a “main coder” to view and code its entirety, while the other coders within its group were assigned a randomly chosen portion of the video consisting of 20% of its length to quadruple-code. 

To conduct this coding, the free and opensource Behavioral Observation Research Interactive Software (BORIS) was used. BORIS was chosen because of its accessibility and its ability to code for both the frequency of behaviors and the duration of time for which behavior was observed. The list of key behaviors was defined in the software’s Ethogram. Coders were able to specify a keyboard key assigned to each behavior to record the corresponding behavior. The recorded events were exported as behavioral binary data with a time interval set as 1 s. That is, all variables were coded as 1 for the presence of that behavior or 0 for lack thereof for each second of the video footage. The descriptions of all behaviors are listed in Table 1.

### 2.4. Inter-Rater Reliability

The total video footage during the 15 observation sessions was 6:27:33 hours, with a mean duration of 25:50 min (min = 5:31 min, max = 49:31 min). As mentioned, the coding was conducted by eight independent student coders who quadruple-coded 20% of each recording. Inter-rater reliability was computed as an intraclass correlation coefficient (ICC) for multiple raters, as discussed in Shrout and Fleiss (1979), for each category of the analysis. For child affect, child behavior, child and caregiver verbalizations, and iPad manipulations, an ICC of 0.88, 0.75, 0.58, and 0.71, *p* < 0.001 for all, was achieved, respectively. Such values are indicative of moderate–good reliability estimates (Koo and Li 2016).

## 3. Results

### 3.1. Descriptive Statistics

Table 2 presents a tetrachoric correlation matrix that depicts the associations between the observed behavioral and affective states of Ryan while he played on the iPad. Key assumptions for the use of the tetrachoric correlation were met. Namely, (a) the underlying distribution of the data was bivariate normal, (b) all variables were linearly related, and (c) the error terms were normally distributed. Ryan’s boredom, defined as his lack of interest in an activity or a slow, unmotivated response to the task at hand, negatively correlated to both his attentiveness (r = −.55) and frustration (r = −.86), and positively correlated to his persistence and exploring (r = .28 and .21, respectively). His attentiveness was moderately associated with his sense of delight (r = −.55) and smiling (r = −.47). The negative correlation indicates that when Ryan was engaged in responding to the app, he did not display any emotion. His frustration positively correlated to his persistence in playing with the iPad (r = .60). While he did not display delight when in a confused affective state (r = −.99), he did seek help from his caregiver (r = .88). There was no significant association between the states of boredom and confusion.

**Figure 1:**
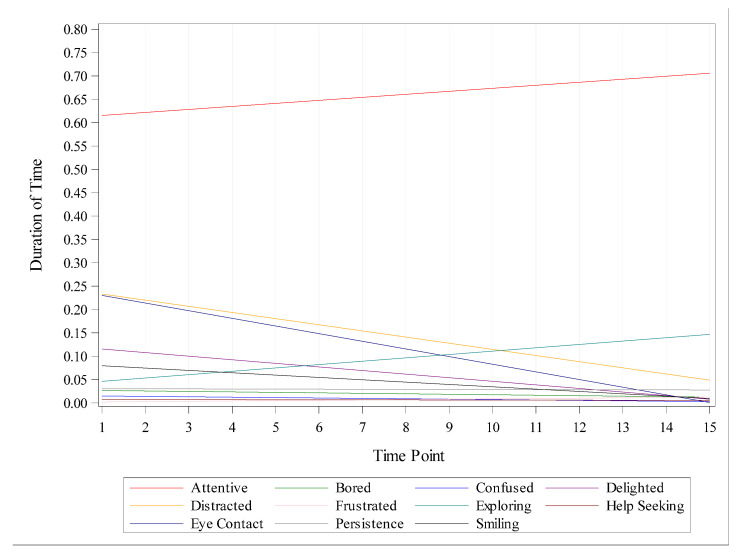
Changes in behavior and affect states over time. Duration of time is measured as the ratio of observed behavior to the recorded footage of each occasion.

Figure 1 depicts the duration of time (measured as the ratio of observed behavior to recorded footage of each occasion) that Ryan exhibited various behavioral and affective states across the 15 timepoints. Notably, Figure 1 is provided only for descriptive purposes; to test for statistical significance, logistic regressions were computed to examine relative change across time. We discovered that as the days passed, Ryan became more attentive, began to explore new games, was less distracted, and had fewer occurrences of eye contact between himself and the caregiver.

The frequency of Ryan’s several iPad manipulations and social interactions during our study are presented in Table 3. During the first few sessions in which Ryan was unfamiliar with iPad navigation, he repeatedly chose to play the same app. He randomly tapped the screen and showed signs of ineffective iPad manipulation (gestures such as hitting the iPad with a palm or fist). He sought attention from the caregiver via verbalizations or eye contact and/or pointed at the source of the problem. As the study progressed, Ryan’s help-seeking behaviors lessened in frequency as his displays of frustration diminished. Furthermore, as his skills developed (e.g., he learned to manipulate the iPad effectively), he began switching between apps more frequently, had fewer questions for his caregiver, and used fewer inappropriate and ineffective manipulations. We believe the increase in his ability to switch between apps is a sign that, as he became more proficient, he got bored. He lost the desire to play on a single app and became interested in exploring the other games that were available to him. As boredom was one of the chosen means to identify Ryan’s bouts of mind-wandering, we believe Ryan engaged in creative problem solving when he grew tired of playing an app, leading to the learned ability to navigate away from his current game to something novel.

Ryan’s caregiver provided direct and indirect commands and raised questions to guide Ryan throughout his play sessions. The caregiver also used verbal praise to encourage Ryan’s exploration and participation in the gaming apps.

### 3.2. Analyses of Observational Data 

Affects and behaviors observed in the video footage were coded such that a code of 1 indicated the presence of behavior for each second of the recording. In order to examine the statistical significance of a relative change in Ryan’s behavioral states, logistic regressions were computed and are presented in Table 4. The results of these analyses indicate that Ryan’s attentiveness, frustration, and exploration increased significantly over time. While Ryan’s boredom and persistence did not significantly change across time, his states of confusion, distraction, and help-seeking behaviors decreased. Smiling and delight also decreased across time. We believe this is evidence that Ryan became more engaged with the DGBL apps since, as previously stated, he did not display signs of positive affect while he was attentive.

Our hypothesis that mind-wandering would lead to positive learning outcomes, defined as Ryan’s ability to creatively problem solve, and gain the ability to manipulate the iPad effectively and its apps, was confirmed by the analysis displayed in Table 5. When exhibited alone, the main effects of boredom and distraction (mind-wandering) both significantly predict attentiveness (β = −2.087 and β = −2.141, *p* < .01, respectively) and do not predict frustration. However, the interaction between boredom and distraction predicts neither attentiveness nor frustration (β = 1.835 and β = .798, *p* = ns, respectively). We also observed that scaffolding in the form of the caregiver’s questions to Ryan predicted Ryan’s attentiveness and frustration (β = .007 and β = −.066, *p* < .01).

We did not examine the statistical significance of changes in Ryan’s iPad manipulations across time. They were coded as the frequency of occurrences across 15 timepoints; thus, we did not have sufficient power to detect significant changes across time.

To better examine Ryan’s learning as evidenced by proper handling and navigation of the device, coded iPad manipulations that were considered appropriate included occurrences of dragging, swiping, and tapping, while inappropriate manipulations included occurrences of hitting the device or using the palm of the hand and/or multiple fingers in an attempt to navigate or play a game. We expected that confusion and attentiveness would predict an increase in navigation proficiency. A linear regression model confirmed this hypothesis (R2 = 0.017, F (3, 23,249) = 129.71, *p* < .01). Namely, confusion (β = 65.952, *p* < .01) and attentiveness (β = 36.62, *p* < .01), and their interaction (β = −145.719, *p* < .01) significantly predicted variance in navigation proficiency. Frustration (β = 81.559, *p* < .01) predicted a significant level of variance in inappropriate navigation (R2 = .008, F (3, 23,252) = 64.28, *p* < .01). Furthermore, Ryan’s help-seeking behavior predicted variation in navigation proficiency (R2 = .069, F (3, 23,252) = 573.72, *p* < .01). While the main effect of help-seeking was significant (β = 69.476, *p* < .01), the interaction was not significant over time (β = −4.423, *p* = 0.164).

## 4. Discussion

This case study with Ryan—a child younger than 3 years of age and thus of an understudied age group in the subject of interest—examined whether mind-wandering is a positive contribution to a child’s learning within the specific context of the use of digital technology when this technology is encountered, in a systematic way, for the first time. We defined learning as the development of app navigation skills and effective iPad manipulations. This study is unique in that typical DGBL studies do not consider media navigation literacy and traditionally prefer to focus on academic material instead (Behnamnia et al. 2020a; Tan et al. 2015). We defined mind-wandering as moments of boredom or distraction and concluded that mind-wandering contributed to positive learning outcomes when Ryan exhibited more attentiveness to the games he played and was able to effectively navigate to an app he liked and within the app itself.

Past studies have shown that a child’s affective state is an important factor related to their learning achievement (Maloney et al. 2015; Valiente et al. 2012). Negative emotions such as boredom, distraction, and confusion, when coupled with attentiveness and persistence, could lead to positive mind-wandering that facilitates creative problem solving, mediating learning (Belton and Priyadharshini 2007; Bonnette et al. 2001; Kort et al. 2001; Pachai et al. 2016; Zhukova et al. 2020). However, the combination of frustration and boredom could lead to negative mind-wandering and a lack of positive learning outcomes (Kort et al. 2001; Maloney et al. 2015; Pachai et al. 2016; Zhukova et al. 2020). These findings are not limited to a specific age range, as experimental designs of mentioned studies included both children of school age as well as adolescents. Importantly, our study focuses on a toddler, widening the age range present in the relevant publications. Although we present a case study, the overall emotional pattern of the engagement of an iPad seems to fit the current literature. 

Ryan’s increased ability to switch between iPad apps is a display of the facets of creative thinking that pertain to dealing with novelty, allowing him to learn how to effectively navigate the digital device (Behnamnia et al. 2020b). The caregiver’s use of verbal praise and other scaffolding encouraged Ryan to explore the available apps and continue to interact with the device (Bonnette et al. 2001). This highlights the importance of child–adult interaction while operating a digital media device. Through the course of the study, we found that Ryan’s states of confusion and distraction, in addition to his help-seeking behaviors, gave way to attentiveness and increased proficiency while using the device. We believe this to be evidence that a child who is allowed to engage in mind-wandering while in the company of a caregiver will be able to engage in creative problem solving, that this will positively contribute to their learning, and that these results can be achieved using a DGBL app on an iPad. Although an iPad itself, perhaps, does not stimulate creativity, it certainly facilities the development of its facets when (1) an iPad is a novel object (i.e., as it was for Ryan at the beginning of the study); (2) skills that are called for by specific apps need to be formed in response to specific situations of dealing with novelty; and (3) a skill has been mastered, and the child gets bored with it, and mind-wandering leads the player to the formulation of novel challenges while discovering and mastering more complex capabilities of an iPad. These observations add to what we currently know about early encounters with digital technology by technology-naïve young children.

Our results also indicate that the duration of time when Ryan was confused and attentive predicted significant variation in his navigation proficiency. This finding corresponds to that of the four phases of learning proposed by Kort et al. (2001) that elucidate a child’s experience of “constructive learning” and “un-learning,” the processes by which they acquire new knowledge while discarding incorrect understanding, respectively.

We highlight that this case study—in times when learning from home is becoming part of the norm—is necessary to bring insights into digital literacy and online education in very early childhood (<3 years old).

## 5. Limitations and Future Research

The findings of this study provide support for the manifestation of positive outcomes during the use of DGBL apps by preschool-aged children. While the research findings on the general effects of media usage on young children are mixed, experts believe that the active role of adult caregivers in the form of questioning and indirect commands can increase these children’s learning, exploration of novelty, critical thinking, and creativity. Thus, mobile devices used in the company of a caregiver may be an effective tool for learning. However, the results of the study should be interpreted with caution, as it was characterized by the following limitations.

First, our study was a case study of a single child, and the generalization of our findings to the population should be applied carefully. Yet, such case studies are very useful as they provide opportunities to investigate underlying principles of an occurrence within a real-life context. Moreover, they are an in-depth investigation of one particular individual, providing a glance into how a young child is handling a tall task of development during his first prolonged exposure to an iPad. A case study allows for the formation of a list of observations that can be verified in future studies. We encourage future researchers to conduct similar studies with groups of children, possibly in both home and school settings, given the changing landscape of how education is delivered.

Second, it is unclear whether or not the app-switching behavior is solely a sign of creative problem solving or whether it is merely a behavior to move away from negative affects, such as frustration and confusion, in order to self-regulate and approach the problem again at a future time. In order to bring clarity to this specific relationship, future research must specifically track task performance and the types of hardships faced in apps. Unfortunately, given the “educational” games that are available, curating a list of evidence-based games may be challenging.

Third, regarding the methods of the study, future endeavors should include a more objective method of affective and behavioral coding. Specifically, researchers interested in the field of DGBL and affective states should inquire about the growing field of affective computing. Affective computing involves systems that detect emotions of use, systems that express what a human would perceive as an emotion, and systems that actually “feel an emotion” (Wu et al. 2016). In other words, affective computing techniques seek to develop algorithms for automatic affect recognition and would involve objective measures, such as autonomic nervous system activity, voice parameters, and facial expressions, complemented with observed behavior in a video (Järvelä et al. 2020). This is the future of the field and would reduce the potential for human errors.

Fourth, it is important to acknowledge that at this young age, it is impossible to elicit a comprehensive and reflective self-report or self-assessment of either learning progress or the corresponding emotions. Therefore, it would be important to evaluate whether the observed emotions correlate with self-reported ones, working, perhaps, with older children. Yet, given the quickly changing reality of child–computer (or digital device) interactions, both with regard to when children start these interactions and for how long they are engaged in them, it might not be possible to identify an age group when children are still naïve to digital devices but already can practice self-evaluation and self-awareness.

While the primary aims of most DGBL apps for young children are to expose them to new material, stimulate their thinking, and add to their knowledge, most are neither research-based nor psychometrically solid. That is, these apps lack empirical evidence to enhance learning and creativity in young children. Developmental benefits reaped from exposure to high-quality children’s media content have been studied at length; however, this research is largely centered on evaluating the content of television programs rather than apps that are accessed on digital devices such as smartphones or tablet/iPad devices (Linebarger et al. 2017). Our case study is a step in obtaining this evidence, but more research is needed to verify that apps that are promoted as “educational” by their designers or retailers truly offer learning benefits to young users. Future educational apps designed for preschool children need to actively involve the child, encourage social interaction with caregivers, and be engaging and meaningful (Hirsh-Pasek et al. 2015).

## Figures and Tables

**Table 1:** Child coding rubric of behavior, affect, verbalization, and iPad manipulation states.

Code Categories	Definition	Duration vs. Frequency	Reference *
**Child Affect**			
Bored	Uninterested in the activity or slow responding to the system without any sign of motivation. Physical description—sitting quietly with minimal engagement with the game and/or the caregiver for a seemingly long period of time. Moving back and forth in his seat, etc.	Duration	Coan and Gottman (2007)
Confused	Difficulty in understanding the material and showing signs of puzzlement. Physical description—frown, pout. Glance for help.	Duration	
Distracted	Looking away or at the caregiver, fidgeting.	Duration	
Frustrated	Visibly angry or agitated state, but at a lower intensity than typical anger. Physical description—lips are frequently thin, with clenched teeth and tightened jaw and neck muscles. A possible sudden increase in voice pitch. Physical agitation (tapping repeatedly).	Duration	Coan and Gottman (2007)
Delighted	Positive affect that is conducive to other positive affects for learning. It is more likely to occur before a flow or surprise state. Physical description—smiling, laughing.	Duration	D’Mello and Graesser (2007)
Surprised	A reaction of sudden happiness to an unanticipated event. Physical description—prominent smiles and loud verbalizations.	Duration	Coan and Gottman (2007)
Attentive/concentrated	Leaning forward and frowning, effortful response.	Duration	
**Child Behaviors**			
Exploring	Browsing the app for something new or of interest unless prompted by the app. Code for this behavior if the app is specific about exploring, ex: flip through pages to trigger animal sounds.	Duration	
Discovery/accident	Unintentional manipulation that leads to discovery with a positive outcome, leading to a better grasp of technology (insight).	Duration	
**Child Behaviors**			
Persistence/repetition	Code for frequency of repetitive actions. When he overcomes a hurdle or switches to the other activity, it signifies the end of his behavior. Typically co-occurs with frustration. Start coding at more than two times of occurrence.	Duration	
Pointing	Pointing with a single finger.	Duration	
Meeting/seeking other’s eye gaze	This behavior consists of trying to look the caregiver in the eyes. The child can be seen actively attempting to do so.	Duration	
Smiling	This is the cheek raiser and lid compressor, as well as the lip corner puller. It is a positive affect.	Duration	Coan and Gottman (2007)
Help-seeking	Stopping out of confusion, distressed vocalizations, looking at the caregiver.	Duration	
Not following app directions/app non-compliance	Dummy code for instances when the child is not doing what the app requires him to do.	Duration	
Not following caregiver’s directions/caregiver’s directions non-compliance	Dummy code for instances when the child is not doing what the caregiver tells him to do. Does not follow *direct* commands from the caregiver.	Duration	
Switches to new app			
**Child Verbalizations**			
Question	The child asks for something using words. It relates to ongoing activity.	Frequency	
Naming	The child labels/names what is in his environment or on the screen according to what is going on.	Frequency	
Vocalization	The child vocalizes as he explores the applications (exclamations, moans, whiny) or says something that cannot be labeled as naming or a question.	Frequency	
Statement repetition	The child repeats the statement the app or caregiver makes.	Frequency	
**Child iPad Manipulation**			
Full hand/multiple fingers	It consists of using a full hand (palms and fingers) or more than one–two fingers at once.	Frequency	Hourcade et al. (2015)
Tap	Mostly a single-finger manipulation. It consists of using one or two fingers to precisely tap on the screen.	Frequency	Hourcade et al. (2015)
Hit	It consists of using one’s hand to hit the iPad.	Frequency	Hourcade et al. (2015)
Drag	This is a manipulation seen in the later stages, as the child becomes more proficient. It consists of using fingers to drag across the screen.	Frequency	Hourcade et al. (2015)
Swipe	It consists of lightly swiping one’s finger(s) across the screen.	Frequency	Hourcade et al. (2015)
Functionally inappropriate	Hits. Any iPad manipulation that cannot be classified as tap, drag, or swipe.	Duration	
Functionally ineffective	All functionally inappropriate manipulations and any tap, drag, or swipe that does not bring the desired outcome.	Duration	
**Caregiver Behaviors**			
Direct command	A direct command is a clearly stated order, demand, or direction in declarative form. The statement must be sufficiently specific to indicate the behavior expected from the child.	Frequency	Eyberg and Robinson (1981)
Indirect command	An indirect command is an order, demand, or direction for a behavioral response that is implied, nonspecific, or stated in question form.	Frequency	Eyberg and Robinson (1981)
Statement	A statement is a declarative sentence or phrase that gives an account of the objects or people in the situation or the activity occurring during the observation.	Frequency	Eyberg and Robinson (1981)
Encouragement	Encouragement is a statement or phrase that expresses approval, appreciation, or positive acknowledgment of the child’s efforts, attributes, or product.	Frequency	Eyberg and Robinson (1981)
Question	A question is a comment expressed in question form. It gives an account of the objects or people in the situation, or the activity occurring during the interaction. This question follows a child’s activity rather than attempting to lead it.	Frequency	Eyberg and Robinson (1981)
Reflective statement	A reflective statement is a statement that repeats all or part of the child’s preceding verbalization. The reflection may be exactly the same words the child said, may contain synonymous words, or may contain some elaboration upon the child’s statement, but the basic content must be the same as the child’s message.	Frequency	Eyberg and Robinson (1981)
Acknowledgement	An acknowledgment is a brief verbal response to the child’s verbalization, behavior that contains no manifest content other than a simple yes or no response to a question, or that communicates a recognition of something the child has said or done with no descriptive content.	Frequency	Eyberg and Robinson (1981)
Irrelevant verbalization	An irrelevant verbalization is a comment or question that pertains to an event, individual, or object that is unrelated to the ongoing activity of the parent or child.	Frequency	Eyberg and Robinson (1981)
Unlabeled praise	Unlabeled praise is a nonspecific verbalization that expresses a favorable judgment on an activity, product, or attribute of the child.	Frequency	Eyberg and Robinson (1981)
Labeled praise	Labeled praise is any specific verbalization that expresses a favorable judgment upon an activity, product, or attribute of the child.	Frequency	Eyberg and Robinson (1981)
Problem solving	Problem solving is a statement, question, or command that invites the child, in an open-ended way, to solve a problem. This could include asking the child to think, plan, organize, and generate ideas, solutions, or consequences. Problem solving is a category we need to double-code for a while, so we do not change our data drastically. Therefore, when problem solving is coded, a question, statement, or command will also be coded.	Frequency	Eyberg and Robinson (1981)
Negative command	A negative command tells the child not to do something. It is a type of critical statement but conveys more specific behavioral information.	Frequency	Eyberg and Robinson (1981)
Re-direction	Redirection is a statement that aims to refocus the attention of the child on a specific task.	Frequency	Eyberg and Robinson (1981)

*Note.* * Empty reference cells indicate behavioral states that were defined by the authors of this study.

**Table 2:** Correlation coefficients depicting the degree of association among observed behavior and affect states.

	1	2	3	4	5	6	7	8	9	10	11
1. Attentive	1.00										
2. Bored	−0.55	1.00									
3. Confused	−0.34	**0.03**	1.00								
4. Delighted	−0.55	−0.15	−0.99	1.00							
5. Distracted	−0.81	−0.12	−0.18	0.59	1.00						
6. Frustrated	−0.50	−0.86	0.18	−0.12	−0.19	1.00					
7. Exploring	0.33	0.21	−0.34	−0.24	−0.51	−0.28	1.00				
8. Help-seeking	−0.22	0.23	0.88	−1.00	**0.01**	0.23	−0.36	1.00			
9. Meeting/seeking Eye Gaze	−0.57	−0.17	**−0.05**	0.53	0.82	−0.38	−0.38	**0.07**	1.00		
10. Persistence repetition	−0.08	0.28	**−0.07**	−0.11	−0.32	0.60	−0.16	**−0.12**	−0.29	1.00	
11. Smiling	−0.47	−0.40	−0.99	0.90	0.50	**−0.09**	−0.28	−0.84	0.60	−0.23	1.00

*Note*. Bold entries denoted are not statistically significant at *p* < .05; all other elements are statistically significant at *p* < .01.

**Table 3:** Total occurrences of behaviors across all timepoints.

Timepoint	1	2	3	4	5	6	7	8	9	10	11	12	13	14	15
	iPad Manipulations														
Switches To new game or app	0	0	0	58	7	1	8	20	47	72	19	30	4	36	93
Drag	64	2	0	18	0	25	4	41	169	163	232	287	31	92	18
Full hand multiple fingers	8	7	1	1	0	8	3	33	1	0	13	18	7	19	0
Hit	0	0	0	1	42	4	4	29	0	0	0	6	1	6	1
Inappropriate	0	19	0	16	37	3	3	50	0	0	3	9	11	38	3
Ineffective	142	89	10	25	234	10	13	193	26	173	49	55	11	145	31
Swipe	58	15	11	15	14	42	66	151	121	102	184	59	97	110	70
Tap	470	302	36	269	289	111	255	348	175	250	175	258	296	271	150
	Caregiver Behaviors														
Acknowledgement	6	5	0	1	10	1	3	23	27	13	13	30	0	20	2
Caregiver question	49	45	10	31	12	16	36	46	32	46	41	46	28	100	18
Direct command	33	8	0	1	2	3	12	15	4	22	11	0	2	28	1
Encouragement	8	7	1	10	1	7	0	2	9	34	4	6	0	18	6
Indirect command	45	13	6	11	3	16	6	19	28	38	7	45	2	10	7
Irrelevant verbalization	24	5	6	30	4	14	15	2	7	27	11	15	7	46	3
Labeled praise	0	0	0	2	3	0	0	1	0	2	0	0	0	8	0
Negative command	2	0	0	1	1	0	0	0	0	1	1	3	0	0	0
Problem solving	18	3	1	2	1	0	0	0	0	15	0	0	1	7	1
Redirection	2	5	3	3	0	5	1	0	0	6	0	0	0	10	0
Reflective statement	17	5	9	3	7	4	4	7	7	17	8	7	2	11	1
Statement	32	27	7	35	4	46	22	45	34	56	21	59	10	46	15
Unlabeled praise	4	20	1	3	0	2	5	15	5	9	10	10	14	23	5
	Child Verbalizations														
Child Question	5	2	0	0	0	0	0	3	1	0	1	4	0	1	0
Naming	29	0	0	6	10	8	10	42	4	11	13	1	6	4	0
Statement Repetition	19	0	0	1	3	9	5	7	9	22	4	8	3	14	0
Vocalization	51	28	17	16	103	22	35	61	34	24	18	54	8	36	8

*Note.* For a description of each behavioral category, please see Table 1.

**Table 4:** Changes in behavior and affect states over time.

	Intercept		β = Time	
Attentive	0.543	(0.031)	0.023	(0.003)
Bored	−3.669	(0.098)	**−0.018**	(0.011)
Confused	−3.902	(0.132)	−0.119	(0.017)
Delighted	−1.894	(0.053)	−0.124	(0.007)
Distracted	−1.226	(0.040)	−0.093	(0.005)
Frustrated	−5.865	(0.204)	0.129	(0.018)
Exploring	−3.129	(0.060)	0.103	(0.006)
Help-seeking	−4.663	(0.170)	−0.055	(0.020)
Eye contact	−1.813	(0.049)	−0.089	(0.006)
Persistence	−3.476	(0.084)	**0.010**	(0.009)
Smiling	−2.101	(0.058)	−0.128	(0.008)

*Note*. Standard errors appear in parentheses. Bold entries denoted are not statistically significant at *p* < .05; all other elements are statistically significant at *p* < .01.

**Table 5:** Logistic regression analysis of predictors of attentive and frustration states.

	Attentive		Frustrated	
	β Estimate(SE)		β Estimate(SE)	
Intercept	1.236	(0.038)	−5.704	(0.000)
Bored	−2.087	(0.228)	**−12.862**	(1006.160)
Distracted	−2.141	(0.144)	**−0.798**	(0.807)
Time	**−0.002**	(0.004)	0.122	(0.000)
Bored × distracted	**1.835**	(1.695)	**0.798**	(3552.010)
Bored × time	−0.099	(0.027)	**−0.122**	(103.530)
Distracted × time	−0.221	(0.024)	−0.012	(0.000)
Bored × distracted × time	**−0.379**	(0.622)	**0.012**	(530.670)
Intercept	0.475	(0.078)	−3.349	(0.380)
Time	−0.034	(0.008)	−0.138	(0.037)
Caregiver question	0.007	(0.002)	−0.066	(0.012)
Caregiver question × time	0.001	(0.000)	0.006	(0.001)

*Note*. Attentive and frustration and their related predictors were analyzed in separate models. Standard errors appear in parentheses. Bold entries denoted are not statistically significant at *p* < .05; all other elements are statistically significant at *p* < .01.

## Data Availability

Raw data can be requested from authors due to privacy or ethical.
